# LncRNA MAGI2-AS3 promotes fracture healing through downregulation of miR-223-3p

**DOI:** 10.1186/s13018-024-04850-5

**Published:** 2024-06-22

**Authors:** Zhiqiang Dong, Bingbing Hu, Shantao Wang, Mingwei Wang, Shengliang Sun, Xinsheng Liu, Danzhi Li, Dengjiang Wu

**Affiliations:** 1 Department of Orthopedics, Xi’an International Medical Center Hospital, Xi’an, 710000 China; 2 Department of Orthopedics, The Affiliated Nanhua Hospital, Hengyang Medical School, University of South China, Hengyang, 421001 China; 3 Spinal Trauma Orthopedics, Yidu Central Hospital of Weifang, No.5168, Jiangjunshan Road, Qingzhou City, Weifang 262500 China; 4 Department of Pediatric, Yidu Central Hospital of Weifang, Weifang, 262500 China; 5 Hand, Foot and Ankle Surgery, Yidu Central Hospital of Weifang, Weifang, 262500 China; 6 Department of Orthopedics, The Fifth Affiliated Hospital of Guangzhou Medical University, No. 621, Gangwan Road, Huangpu District, Guangzhou, 510700 China

**Keywords:** MAGI2-AS3, Fracture healing, Delayed healing, miR-223-3p, Osteoblast

## Abstract

**Background:**

Long non-coding RNAs (LncRNAs) are recognized as a pivotal element in the processes of fracture healing and the osteogenic differentiation of stem cells. This study investigated the molecular mechanism and regulatory significance of lncRNA MAGI2-AS3 (MAGI2-AS3) in fracture healing.

**Methods:**

Serum levels of MAGI2-AS3 in patients with normal and delayed fracture healing were verified by RT-qPCR assays. The predictive efficacy of MAGI2-AS3 for delayed fracture healing was analyzed by ROC curve. Osteogenic markers were quantified by RT-qPCR assays. MC3T3-E1 cell viability was detected using CCK-8 assay, and flow cytometry was utilized to measure cell apoptosis. The dual-luciferase reporter gene assay was used to determine the targeted binding between MAGI2-AS3 and miR-223-3p.

**Results:**

Serum MAGI2-AS3 expression was decreased in patients with delayed fracture healing compared with patients with normal healing. Elevated MAGI2-AS3 resulted in an upregulation of the proliferative capacity of MC3T3-E1 cells and a decrease in mortality, along with increased levels of both osteogenic markers. However, after transfection silencing MAGI2-AS3, the trend was reversed. Additionally, miR-223-3p was the downstream target of MAGI2-AS3 and was controlled by MAGI2-AS3. miR-223-3p mimic reversed the promoting effects of MAGI2-AS3 overexpression on osteogenic marker levels and cell growth, and induced cell apoptosis.

**Conclusion:**

The upregulation of MAGI2-AS3 may expedite the healing of fracture patients by targeting miR-223-3p, offering a novel biomarker for diagnosing patients with delayed healing.

## Introduction

Fractures are a frequently encountered condition in clinical settings, with tibia fractures in adults being particularly prevalent [1]. The process of fracture repair is intricate and protracted, and delayed healing imposing substantial burdens on patients [2]. It involves not only physical and emotional distress but also entails considerable financial expenditure. Given the vulnerability of soft tissues and poor blood flow in the lower leg, tibial fractures carry a considerable risk of delayed or non-union [3]. Meanwhile, factors such as inadequate nutrition, infection, and blood circulation disruption serve as pivotal indicators of delayed fracture healing [4]. Calcitonin was found to potentially accelerate fracture healing in studies of means of fracture healing and that intermittent pneumatic compression care also aids in soft tissue healing [5, 6]. Moreover, low-intensity pulsed ultrasound was considered as an effective and less invasive treatment for patients with delayed healing [7]. Notably, the functional activity of osteoblasts has been implicated in fracture healing, and their proliferation and differentiation of osteoblasts are essential for the formation of new bone [8]. Furthermore, as osteoblasts become more metabolically active, there is a rise in the levels of osteogenic markers, which serve as indirect indicators to monitor the progress of fracture healing [9]. By understanding how to regulate osteoblast function and monitor osteogenic marker changes, we can gain a better understanding of the fracture healing process. Hence, the pursuit of convincing biomarkers to explore the mechanism of fracture healing holds significant practical value for the healing and monitoring of individuals suffering from fractures.

LncRNAs have been established as participants in the regulation of the cell cycle, chromatin modification and various life activities in vivo, as supported by extensive epigenetic and molecular studies [10]. In the recent years, lncRNAs have become a significant focus in bone metabolism research. For instance, Lei et al. discovered that lncRNAs contribute to bone destruction in rheumatoid arthritis by mediating cell autophagy [11]. Xue et al. realized the efficacy of lncRNA SNHG14/miR-493-5p/Mef2c regulatory network in alleviating osteoporosis [12]. Interestingly, MAGI2-AS3 targeting miR-374b-5p ameliorated the injury and inflammatory response of nucleus pulposus cells in intervertebral disc degeneration [13]. MAGI2-AS3 is a lncRNA transcribed from the antisense strand of MAGI2, located on chromosome 7q21.11, and was found to be abnormally expressed in human tumors and neurological disorders [14–16]. However, the specific relationship between MAGI2-AS3 and fracture healing has not been widely acknowledged.

On this basis, the present study reflected the diagnostic potential of MAGI2-AS3 in delayed fracture healing by quantitatively measuring its levels in patients with normal and delayed fracture healing. Additionally, the molecular mechanism of MAGI2-AS3 in fracture healing was revealed by in vitro cellular assays, aiming to seek novel therapeutic strategies for fracture healing.

## Materials and methods

### Inclusion of patients

The subjects of the study were 119 patients, all over 18 years old, with unilateral tibial fractures. These patients underwent surgical treatment from November 2022 to September 2023 at Yidu Central Hospital of Weifang. Any patients with previous history of fracture, osteoporosis, or fractures at other sites were excluded from the study. The experimental protocol was reviewed and approved by the Ethics Committee of Yidu Central Hospital of Weifang, and all the participats provided their informed consent.

### Grouping of patients

Patients were monitored for 4 months post-operation to observe fracture healing. They were categorized into two groups based on prognosis: the normal healing group (*n* = 63) and the delayed healing group (*n* = 56). All patients in the normal healing group displayed bone scabs formation at the tibial fracture site, and their activities returned to normal without pain [17]. In contrast, the delayed healing group exhibited a noticeable gap between the fracture ends on X-ray images, with no continuous callus and dense sclerotic bone.

### Collection of serum samples

Blood samples were collected from the patients and centrifuged (2000 rpm, 15 min) after standing for 10 min. The upper layer of serum was subsequently collected and cryopreserved.

### Cultivation of cells

MC3T3-E1 cells were purchased from the RIKEN Cell Bank (Tsukuba, Japan), and cultured in DMEM medium, which contains 10% FBS and 1% penicillin-streptomycin. The cells were seeded and placed in an incubator set to 37 °C, with an environment of 95% air and 5% CO_2_.

### Transfection of cells

Transfection vectors pcDNA3.1-MAGI2-AS3, si-NC, si-MAGI2-AS3, mimic NC, and miR-223-3p mimic were synthesized by RiboBio (Guangzhou, China). Each of these vectors was mixed with the transfection reagent Lipofectamine 2000 (Invitrogen, Carlsbad, USA), and then added to MC3T3-E1 cells in the logarithmic phase for transfection assay. The medium was replaced every 6 h, and the cells were harvested 24 h after transfection.

### RT-qPCR reaction

Total RNA was extracted from the serum and cells for testing using the TRIzol reagent. After confirming the RNA concentration, reverse transcription was executed using the HiFiScript cDNA Synthesis Kit (Cwbiotech, China). Once the cDNA was obtained, quantitative PCR analysis was conducted on an MX3000p Real-time PCR instrument via SYBR premix Ex Taq II Kit (Takara Bio, Japan). With β-actin and U6 as reference genes, the MAGI2-AS3, miR-223-3p, ALP mRNA, OC mRNA and Runx2 mRNA expression were calculated using the 2^−ΔΔCt^ method.

### CCK-8 assay

The transfected MC3T3-E1 cells were inoculated into 96-well plates at a concentration of 2 × 10^3^ cells, and CCK-8 solution was added after incubation for 0, 24, 48, and 72 h. Incubation was terminated after 4 h at 37 °C, and the cells were then shaken in a shaker (Thermo Scientific, USA) for 5 min. Absorbance values were determined at 450 nm using a microplate reader (Thermo Scientific, USA).

### Apoptosis assay

MC3T3-E1 cells treated with transfection were gathered and rinsed in PBS. Annexin V-FITC and PI reagents (Annexin V-FITC/PI Kit; Biotech, China) were added to the cells and incubated for 10 min in the dark. Following this, apoptotic cell counts were assessed via flow cytometry.

### Dual-luciferase reporter assay

Fragments of MAGI2-AS3 containing miR-223-3p binding sites were inserted into the pmiRGLO vector to create either wild-type (WT) or mutant-type (MUT) MAGI2-AS3 fragments. These vectors were co-transfected with either a mimic NC or a miR-223-3p mimic into MC3T3-E1 cells. The luciferase activity was assessed 48 h post-transfection using a dual-luciferase reporter assay system (Promega, Wisconsin, USA).

### Statistical analysis

SPSS 21.0 software was utilized to process the experimental data. Intergroup comparisons of clinical indicators between the two groups of patients were performed by chi-square test. The risk factors of delayed fracture healing were evaluated by Binary logistic regression. Levels of MAGI2-AS3 and miR-223-3p were compared between groups through t-test and ANOVA. The predictive value of MAGI2-AS3 expression in patients with delayed fractures was evaluated by ROC curve. The target and binding sites of MAGI2-AS3 were predicted by ENCORI and lncRNASNPV3 online databases. *P* < 0.05 was believed a statistically significant difference.

## Results

### Serum MAGI2-AS3 was lowly expressed in patients with delayed healing

**Fig. 1:**
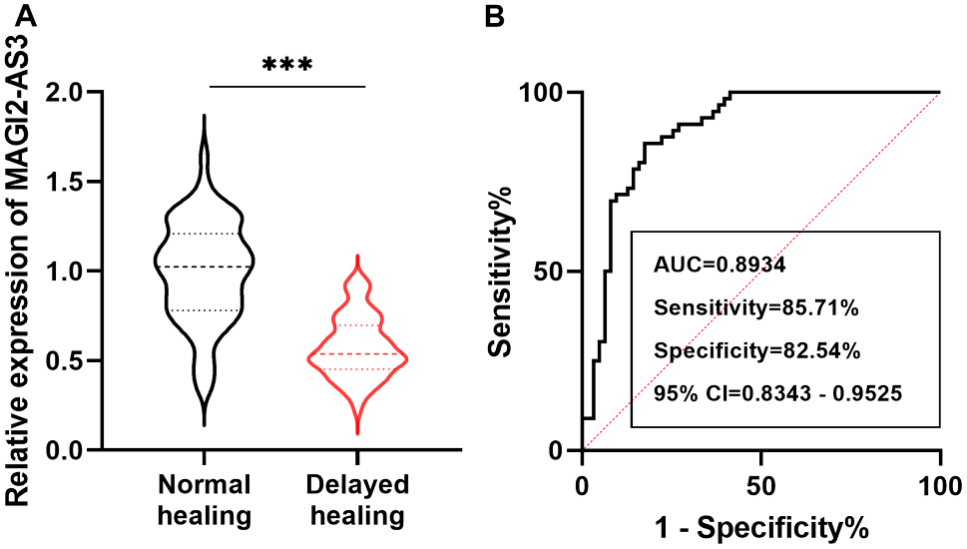
Analysis of MAGI2-AS3 expression and diagnostic value in included patients with tibial fractures. **A**. Serum MAGI2-AS3 levels were decreased in delayed healing patients compared to normal healing patients. **B**. AGI2-AS3 had high diagnostic ability in predicting delayed fracture healing in patients. *** *P* < 0.001, compared with normal healing

The RT-qPCR results of indicated that serum MAGI2-AS3 was downregulated in patients with delayed fracture healing compared to normal fracture healing (Fig. 1A, *P* < 0.001). Furthermore, the ROC curve elucidated that the sensitivity and specificity of MAGI2-AS3 in predicting delayed healing were 85.71% and 82.54%, with an AUC of 0.8934 (95% CI = 0.8343–0.9525), suggesting that abnormal MAGI2-AS3 levels may be associated with delayed fracture healing (Fig. 1B).

### General information of the subjects and the clinical potential of MAGI2-AS3

By analyzing and comparing the clinical data of patients with normal and delayed fracture healing, it is observed in Table 1 that there were no significant differences in age, BMI, gender, fracture side, severity of fracture, osteosynthesis method, smoking and drinking habits between the two groups (*P* > 0.05). Moreover, MAGI2-AS3 may be a factor influencing delayed fracture healing, as revealed by Binary logistic regression used for assessing risk indicators of delayed healing (Table 2, *P* < 0.001).

**Table 1:** Analysis of general information on patients with fracture healing

Indicators	Normal healing (*n* = 63)	Delayed healing (*n* = 56)	*P*
Age (year)	48.92 ± 13.28	47.16 ± 16.08	0.514
BMI (kg/m^2^)	23.70 ± 3.42	23.89 ± 3.26	0.749
Gender			0.745
Female	33	31	
Male	30	25	
Fracture side			0.517
Left	30	30	
Right	33	26	
Severity of fracture			0.458
Complete fracture	25	26	
Incomplete fracture	38	30	
Osteosynthesis method			0.160
Open reduction	29	33	
Losed reduction	34	23	
Smoking			0.693
No	27	22	
Yes	36	34	
Drinking			0.635
No	21	21	
Yes	42	35	

**Table 2:** Binary logistic regression analysis of clinical indicators in patients with fracture healing

Indicators	OR	95% CI	*P*
MAGI2-AS3	0.026	0.008–0.086	< 0.001
Age	1.470	0.544–3.974	0.447
BMI	1.893	0.696–5.147	0.211
Gender	1.731	0.625–4.798	0.291
Fracture side	1.655	0.593–4.616	0.336
Osteosynthesis method	1.974	0.717–5.436	0.188
Smoking	1.634	0.554–4.819	0.374
Drinking	1.125	0.384–3.296	0.831

### Regulation of osteoblast activity by abnormal expression of MAGI2-AS3

**Fig. 2:**
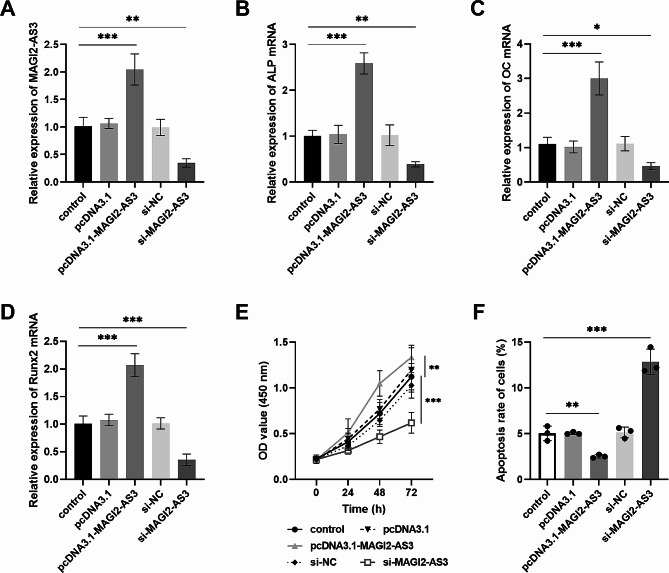
Regulation of osteoblast reproduction by up or down-regulation of MAGI2-AS3 expression. **A**. Transfection of pcDNA3.1-MAGI2-AS3 resulted in elevated levels of MAGI2-AS3 in cells, whereas the MAGI2-AS3 expression was suppressed by si-MAGI2-AS3 transfected. **B-D**. Effect of transfection with abnormal levels of MAGI2-AS3 on osteogenic marker levels. **E-F.** Regulation of osteoblast proliferation and apoptosis by aberrant expression of MAGI2-AS3. * *P* < 0.05, ** *P* < 0.01, *** *P* < 0.001, compared with control

In the study exploring the molecular mechanism of MAGI2-AS3 in fracture healing, the present study transfected pcDNA3.1-MAGI2-AS3 or si-MAGI2-AS3 into osteoblast MC3T3-E1 to upregulate or downregulate the content of MAGI2-AS3 (Fig. 2A). Subsequently, the examination of osteogenic marker levels revealed that increasing MAGI2-AS3 enhanced the ALP (Fig. 2B), OC (Fig. 2C), and Runx2 (Fig. 2D) levels. Conversely, dysregulation of MAGI2-AS3 suppressed these levels. MC3T3-E1 cell activity assay also revealed that prominently expressed MAGI2-AS3 significantly improved cell proliferation and reduced cell apoptosis, while silencing MAGI2-AS3 inhibited cell growth and induced cell apoptosis in Fig. 2E and F. These findings imply that overexpression of MAGI2-AS3 may promote fracture healing.

### MiR-223-3p is the direct target of MAGI2-AS3

**Fig. 3:**
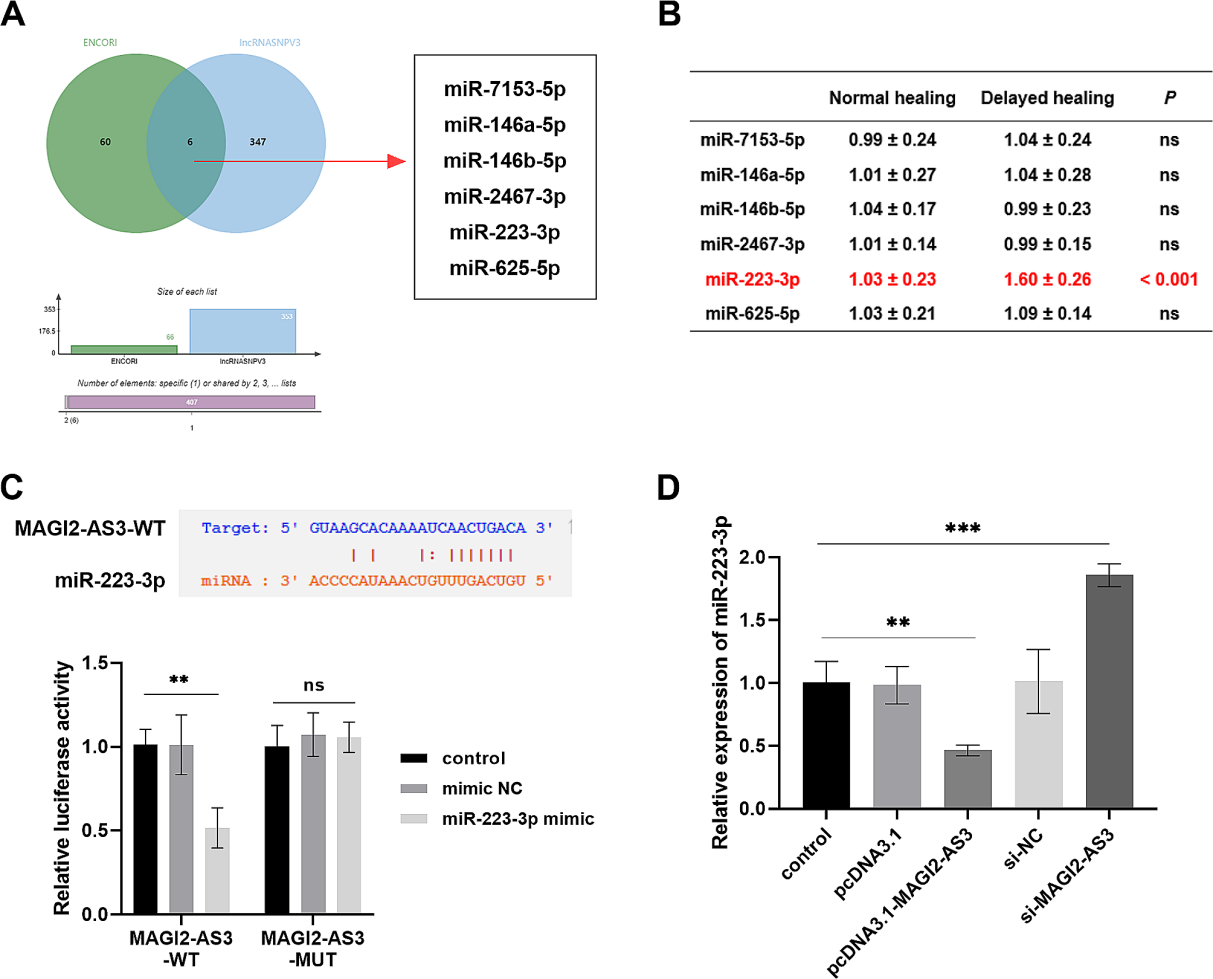
MAGI2-AS3 directly sponges miR-223-3p. **A**. The downstream target genes of MAGI2-AS3 predicted by the bioinformatics website are shown in the Venn diagram. **B**. Expression of different miRNAs in the included fracture patients. **C**. The targeted combination of MAGI2-AS3 and miR-223-3p was analyzed by bioinformatics and dual-luciferase reporter gene detection. **D**. Regulation of miR-223-3p expression by transfection of abnormal levels of MAGI2-AS3. ^**ns**^*P* < 0.05, ** *P* < 0.01, *** *P* < 0.001, compared with control

A search of the online databases ENCORI and lncRNASNPV3 revealed that miR-7153-5p, miR-146a-5p, miR-146b-5p, miR-2467-3p, miR-223-3p, and miR-625-5p are downstream targets of MAGI2-AS3 (Fig. 3A). RT-qPCR assays then validated that serum miR-223-3p was enriched in patients with delayed healing, as determined by the levels of the above miRNAs (Fig. 3B, *P* < 0.001). Further confirmation of the targeting relationship between MAGI2-AS3 and miR-223-3p came from bioinformatics software prediction. This predicted the presence of binding sites between MAGI2-AS3 and miR-223-3p. Additionally, luciferase activity was remarkably decreased after co-transfection of miR-223-3p mimic with MAGI2-AS3-WT (Fig. 3C, *P* < 0.01). After transfection of pcDNA3.1-MAGI2-AS3 or si-MAGI2-AS3 in MC3T3-E1 cells, the expression of miR-223-3p decreased or increased (Fig. 3D). These results imply that MAGI2-AS3 directly sponges and negatively regulates miR-223-3p expression in fracture healing.

### Upregulation of mir-223-3p attenuated the influence of elevated MAGI2-AS3 on osteoblasts

**Fig. 4:**
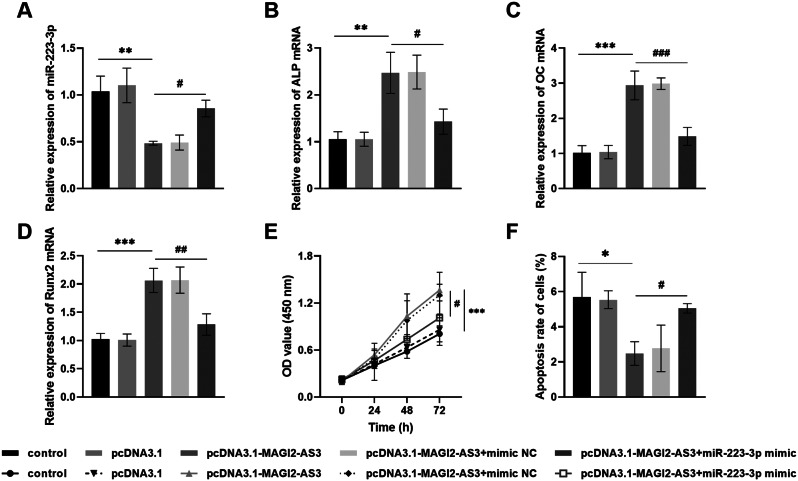
Mediation of miR-223-3p mimic on osteoblasts. **A**. Upregulation of miR-223-3p expression in osteoblasts after transfection with pcDNA3.1-MAGI2-AS3 + miR-223-3p mimic. **B-D**. High levels of MAGI2-AS3 improved the levels of osteogenic markers, while co-transfection with overexpression of miR-223-3p reversed this promoting effect. **E-F.** pcDNA3.1-MAGI2-AS3 accelerated osteoblast growth and suppressed apoptosis, while miR-223-3p mimic had the opposite effect. * *P* < 0.05, ** *P* < 0.01, *** *P* < 0.001, compared with control; ^**#**^*P* < 0.05, ^**##**^*P* < 0.01, ^**###**^*P* < 0.001, compared with pcDNA3.1-MAGI2-AS3

We further investigated the fracture healing mechanism by co-transfecting of pcDNA3.1-MAGI2-AS3 and miR-223-3p mimic in MC3T3-E1 cells. In Fig. 4A, MAGI2-AS3 elevation suppressed miR-223-3p expression, but miR-223-3p levels were restored after its upregulation. Concurrently, transfection with pcDNA3.1-MAGI2-AS3 upregulated the osteogenic markers ALP, OC, and Runx2 levels, whereas miR-223-3p mimic diminished their expression (Fig. 4B and D). In addition, MAGI2-AS3 overexpression promoted cell growth and reduced cell apoptosis. However, the combination of pcDNA3.1-MAGI2-AS3 and miR-223-3p mimic reversed MAGI2-AS3’s beneficial effects on cell activity and stimulated cell apoptosis (Fig. 4E and F). Therefore, the outstanding expression of MAGI2-AS3 may mediate the activity of osteoblasts by adsorbing miR-223-3p, thereby accelerating fracture healing.

## Discussion

Fracture healing is a complex biological process that involvese aspects like inflammatory response, neovascularization, and the generation of cartilage and bone tissue. Factors such as a patient’s nutritional status, circulatory status, metabolic level, and age also impact the speed and quality of fracture healing [18]. For effective healing, it’s essential to ensure the stability of the broken bone and maintenance of sufficient soft tissue coverage to provide an adequate blood supply, which helps to activate endogenous stem cells to repair damaged tissue [19]. Given the distinctive positioning of the tibia and the characteristics of the associated tissues, patients may experience delayed healing. Therefore, delving into the mechanism of lncRNAs in fracture healing holds practical value.

MAGI2-AS3 is a novel lncRNA that has been observed to have dysregulated expression in human diseases and biological processes by multiple reports [16, 20]. MAGI2-AS3 appears to related to the regulation of expression of the MAGI2 gene, and the protein encoded by MAGI2 participate in processes such as cell signaling and cell adhesion [21]. Silencing MAGI2-AS3 mediated HSPA8 protein level to delay cell senescence, which may assist cells in anti-aging regulation [22]. Additionally, it was observed that MAGI2-AS3 is poorly expressed in breast cancer [23], hepatic cancer [24], and esophageal cancer [25]. In the study on the mechanism of MAGI2-AS3, it inhibited inflammatory responses by downregulating the levels of miR-374b-5p and alleviated myeloid cell damage and outer matrix degradation by promoting IL-10 levels [13]. MAGI2-AS3 was determined to be downregulated in leukemic stem cells and inhibited cell renewal by upregulating LRIG1, thereby improving the deterioration of acute myeloid leukemia [26]. More critically, MAGI2-AS3 was revealed to be downregulated in patients with intervertebral disc degeneration, whereas elevated levels of MAGI2-AS3 suppressed Fas ligand expression and mediated the nucleus pulposus cells [27]. And upregulation of MAGI2-AS3 affected intervertebral disc therapy by repressing the levels of FasL gene and protein [28]. Consequently, we hypothesized that MAGI2-AS3 may be engaged in the mechanism of fracture healing. To test this hypothesis, 119 patients with tibial fractures were enrolled in this study, and among the total number of participants, there were 63 patients with normal healing fractures and 56 patients with delayed healing. The results proposed that serum MAGI2-AS3 expression was reduced in the delayed healing group, and low level of MAGI2-AS3 had a high diagnostic significance for distinguishing patients with delayed fracture healing, suggesting its possible involvement in the pathological process of fracture healing.

As healing proceeds, the number of osteoblasts increased progressively, and the differentiation and activity of the cells contribute to the acceleration of new bone formation [29]. Biomarkers of osteoblast activity and function, like ALP, OC, and Runx2, increase during fracture healing, reflecting osteoblast activity and new bone formation [30]. Our study elucidated that enhancing MAGI2-AS3 expression significantly increased the levels of osteogenic markers ALP, OC, and Runx2, whereas inhibiting MAGI2-AS3 reduced their levels. Furthermore, overexpression of MAGI2-AS3 enhanced the growth capacity of the osteoblast MC3T3-E1 and repaired apoptosis, while silencing MAGI2-AS3 affected cell viability and induced apoptosis. These findings confirmed that MAGI2-AS3 contributes to fracture healing when it is increased, and reduced MAGI2-AS3 level may lead to delayed fracture healing.

In addition to lncRNAs, miRNAs have also demonstrated their involvement in musculoskeletal diseases. For instance, research conducted by Lorenzo and colleagues highlighted the promising role of miRNAs in the clinical management of tendon injuries [31]. miR-223-3p is located on the X chromosome, which is identified as a pivotal regulator in bone diseases [32]. In a study by Long et al. the miR-223-3p/FOXO3 axis promoted osteogenic differentiation of bone marrow mesenchymal stem cells by elevating autophagy [33]. Dong et al. proposed that miR-223-3p impacts chondrocyte activity and inflammatory response and may be a target of sinomenine in treating osteoarthritis [34]. Furthermore, miR-223-3p was enriched for expression in osteoporosis patients skeletal muscle injured mice [35, 36]. In this research work, the bioinformatics website verified that miR-223-3p is a downstream target of MAGI2-AS3 and that there are targeted binding sequences between them. miR-223-3p was prominently expressed in patients with delayed fracture healing and was inversely regulated by MAGI2-AS3. Correspondingly, miR-223-3p levels were significantly elevated in fracture patients compared to healthy individuals and were involved in fracture healing therapy by mediating FGFR2 [37]. Additionally, transfecting cells with miR-223-3p mimic lessened the enhancingeffect of upregulated MAGI2-AS3 on osteogenic markers, and revived the increase in osteoblast viability. Furthermore, the function of small interfering RNAs (siRNAs) cannot be ignored. siRNAs was found to be involved in the therapeutic process of tendon repair, rheumatoid arthritis and osteoporosis by mediating gene regulation [38–40]. The specific pathological process and mechanism are also the content of our subsequent mining.

## Conclusion

Briefly, the study examined the regulatory capacity of MAGI2-AS3 in fracture healing. MAGI2-AS3 expression was decreased in patients with delayed healing, and it regulated osteoblast activity of osteoblasts through sponge miR-223-3p. An increase in MAGI2-AS3 levels enhanced osteoblast proliferation, diminished cell apoptosis, and facilitated fracture healing in patients, while miR-223-3p mimic reversed the effect of MAGI2-AS3 overexpression. These discoveries offer novel insights for enhancing fracture healing and the accurate prediction of delayed healing.

## Data Availability

Corresponding authors may provide data and materials.
